# Maximizing Electrochemical Information: A Perspective on Background-Inclusive Fast Voltammetry

**DOI:** 10.1021/acs.analchem.3c04938

**Published:** 2024-04-10

**Authors:** Cameron S. Movassaghi, Miguel Alcañiz Fillol, Kenneth T. Kishida, Gregory McCarty, Leslie A. Sombers, Kate M. Wassum, Anne Milasincic Andrews

**Affiliations:** 1 Department of Chemistry and Biochemistry, University of California, Los Angeles, Los Angeles, California 90095, United States; 2 Interuniversity Research Institute for Molecular Recognition and Technological Development, Universitat Politècnica de València-Universitat de València, Camino de Vera s/n, Valencia 46022, Spain; 3 Department of Translational Neuroscience, Wake Forest School of Medicine, Winston-Salem, North Carolina 27101, United States; 4 Department of Neurosurgery, Wake Forest School of Medicine, Winston-Salem, North Carolina 27101, United States; 5 Department of Chemistry, North Carolina State University, Raleigh, North Carolina 27695, United States; 6 Comparative Medicine Institute, North Carolina State University, Raleigh, North Carolina 27695, United States; 7 Department of Psychology, University of California, Los Angeles, Los Angeles, California 90095, United States; 8 Brain Research Institute, University of California, Los Angeles, Los Angeles, California 90095, United States; 9 Integrative Center for Learning and Memory, University of California, Los Angeles, Los Angeles, California 90095, United States; 10 Integrative Center for Addictive Disorders, University of California, Los Angeles, Los Angeles, California 90095, United States; 11 Department of Psychiatry and Biobehavioral Science, University of California, Los Angeles, Los Angeles, California 90095, United States; 12 Hatos Center for Neuropharmacology, University of California, Los Angeles, Los Angeles, California 90095, United States

## Abstract

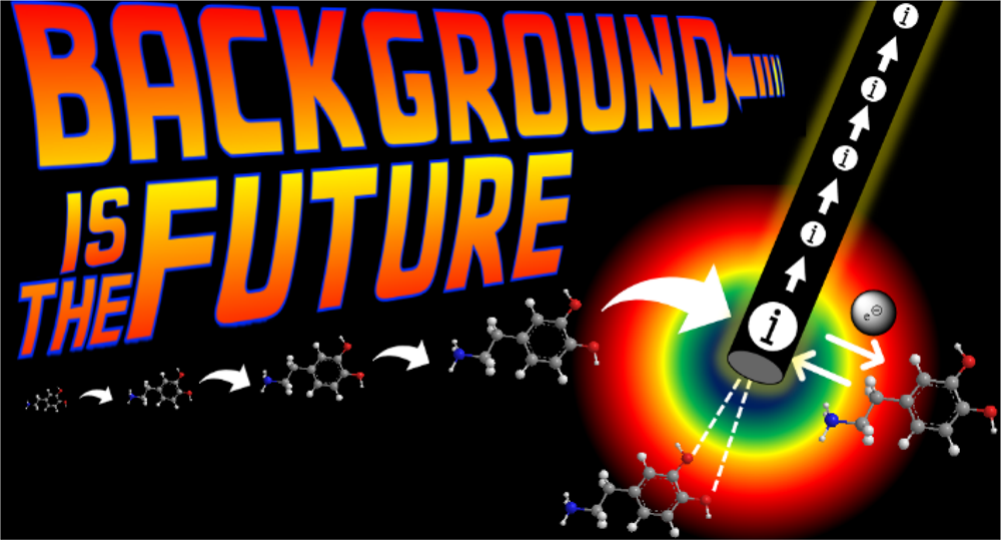

This perspective encompasses a focused review of the literature leading to a tipping point in electroanalytical chemistry. We tie together the threads of a “revolution” quietly in the making for years through the work of many authors. Long-held misconceptions about the use of background subtraction in fast voltammetry are addressed. We lay out future advantages that accompany background-inclusive voltammetry, particularly when paired with modern machine-learning algorithms for data analysis.

## Introduction

Background subtraction for bioanalytical voltammetry was first reported in the 1980s.^1,2^ Its purpose, as originally described, was to increase the signal-to-noise ratio or otherwise aid in visualizing small faradaic currents (tens of nanoamperes (nA) or less) produced by neurotransmitter release associated with biological stimulus events. Small, analyte-related currents occur amid large capacitive currents (hundreds of nA) produced by the high scan rates used in fast-scan cyclic voltammetry (FSCV). For almost four decades, background subtraction has been *de rigueur* in fast voltammetry (e.g., FSCV,^3,4^ FSCAV,^5^ FCSWV^6^). Today, even the smallest stimulus peaks associated with endogenous transients can be readily identified by fast voltammetry and related techniques with modern data acquisition and analysis capabilities.^7−10^

While often discarded, background currents can be sources of electrochemical information for analyte identification.^11−15^ Moreover, retaining background currents overcomes a pitfall associated with fast voltammetry—the inability to use the same waveform to measure basal neurotransmitter levels and stimulus-related events contemporaneously.

“The study of basal levels of neurotransmitters and their dynamics requires a means of isolating the portion of the background current arising from neurotransmitter redox reactions.”—Johnson et al. 2018^60^

In this perspective, we delve into the practice of background subtraction, developed during a period when electronic sampling and computational capabilities were less advanced. We outline the advantages of forgoing background subtraction, at least under some circumstances. While we frame this perspective in the context of neurochemical detection, the ideas developed are relevant to voltammetry for other types of analytes.

Background currents are composed of faradaic and nonfaradaic contributions and noise (e.g., electrical, environmental). In neurochemical studies, the background current is represented by a voltammogram relative to a paired experimental stimulus event and is commonly determined within a 30–90 s recording window immediately before event recording. Background voltammograms are often averages of consecutive prestimulus scans (e.g., 5–10 voltammograms), which improve the signal-to-noise ratio for background-subtracted traces. The process of background subtraction produces differential measurements (i.e., determinations of current after vs before a defined time point). The applicability of the defined background current relative to the length of the recording window depends on signal stability and other factors discussed below.

Seminal papers on background subtraction explicitly stated that its purpose was to facilitate peak visualization and calibration when manual peak selection and integration were often required.^1^ Based on its original purpose, we suggest background subtraction may no longer be needed. Moreover, in some cases, information inherent in background currents can be used to improve analyte identification and quantitation, particularly for multianalyte detection.

## Pitfalls Associated with Background Subtraction

We suspect that background subtraction remains prevalent partly because the term is somewhat of a misnomer. That is, background subtraction is not background correction. Background subtraction cannot remove dynamic nonspecific current contributions. Thus, it may not result in selective analyte current. Nonetheless, background subtraction is often employed with the underlying implication that analyte-specific faradaic current changes remain after stimulus events.^18^ During the recording period after a stimulus, however, the concentrations of nontarget analytes (i.e., interferents) and ions at the electrode surface change in response to the stimulus. Some of these species are redox active (e.g., neurotransmitter metabolites). As such, they contribute to nonspecific changes in faradaic current. Other species, while not electrochemically active, affect electrical double-layer behavior and thus contribute to changes in nonfaradaic current. While noncharged, nonelectroactive species (e.g., glucose) do not directly affect current responses in physiological media,^19^ such species can impact electrode surface accessibility.

In neurochemistry, any type of stimulus contributes to nonspecific current changes, including stimuli delivered *in vivo* (e.g., behavioral stimuli), *ex vivo* (e.g., tissue slice electrical or optical stimulation), or *in vitro* (e.g., single-cell analyses involving spritzing with secretagogues). Changes in the concentrations of charged molecules and ions, whether electroactive or not, affect capacitive currents due to uncompensated resistance. Fluctuations occur in the concentrations of ions inherent in the processes underlying neurotransmitter release and reuptake (e.g., pH shifts and ion changes tied to action potentials, Na^+^/K^+^ ATPase activity, and active transport). Background subtraction cannot correct for the effects of these dynamic processes.^20^ A few clever yet cumbersome approaches to correct partially for nonspecific current dynamics exist, as studied by Johnson and colleagues.^21^

“FSCV data analysis typically employs digital subtraction of the background using the current measured before the neurobiological phenomena of interest. This method is effective for signal isolation given background stability. However, if neurotransmitter release is accompanied by factors that affect the background, the subtracted data contain artifacts.”—Johnson et al. 2017^21^

In best-case scenarios, background subtraction preserves much of the poststimulus neurotransmitter-related data. However, background subtraction can remove relevant, or even introduce irrelevant, features. Wosiak and co-workers have investigated these effects.^18^

“Due to the existence of induced charging currents, the capacitive contribution to the total current is different from the capacitive current measured in the absence of electroactive species...Consequently, the conventional background subtraction method may be inaccurate in these situations.”—Wosiak et al. 2020^18^

Additionally, background subtraction cannot correct for drift, which is dynamic during FSCV recording periods.^22^ Several papers address the drift that remains after background subtraction.^23,24^ While background subtraction can improve temporal current responses for short recording periods (e.g., <90 s), this approach assumes that drift is due solely to capacitive current instability that does not change measurably after the background is determined and over the recording period.^25^ Newer, more effective approaches to deal with drift are aimed at extending the time frame of FSCV recordings.^23,24,26,27^ However, as also noted by Johnson, the chemistry at the electrode surface is complicated and dependent on the surrounding microenvironment.^21^

“Interactions with the carbon surface, through either adsorption or involvement in surface reactions, may alter these responses and contribute to the background-subtracted voltammograms. Indeed, nonfaradaic and faradaic currents have been seen in background-subtracted voltammograms taken during pH changes.”—Johnson et al. 2017^21^

Background currents in voltammetry are inherently dynamic, which is at the root of these misconceptions. Changes occur in the background signal, defined as the current generated by everything except the analyte of interest, even on the time scale that background subtraction is employed. Background signals are impacted by changes in electrode surface chemistries (e.g., analyte or interferant adsorption, electrode surface group oxidation, biofouling) and by changes in ion concentrations associated with action potentials, transporter-mediated reuptake ([Na^+^], [K^+^]), and exocytosis ([H^+^], [Ca^2+^]). Subtracting the background preceding stimulus events, although previously useful for improving peak identification, ignores these dynamic processes by incorrectly assuming a static microenvironment during the user-defined recording periods typical in FSCV (e.g., 30–90 s). As we propose, background subtraction can also reduce predictive accuracy in certain cases. Indeed, previous studies have shown that background changes can lead to misinterpretations of biological findings.^28,29^

This is not to say that all voltammetry studies using background-subtracted approaches are invalid, nor that background-inclusive data are superior in all cases. Voltammetry would not have advanced without background subtraction. There is likely a “Goldilocks zone” where background-subtracted and nonbackground-subtracted interpretations largely agree.

We simply advocate reconsidering the significant information included in the background current. Data analyses using background subtraction vs. background inclusion are not mutually exclusive; one can analyze and compare both approaches using the same data. However, as background-inclusive fast voltammetry has emerged relatively recently in neuroscience compared to its predecessor, few studies have compared these approaches directly.^11,30^ Regardless of the approach employed, data must always be interpreted with caution. For in the words of statistician George Box, all models are wrong, but some are useful.^130^

Regardless of whether background subtraction is used or not, there are pervasive issues for *in vivo* voltammetry. Perhaps the most significant is the difficulty in generalizing *in vitro* calibration data, including calibration parameters estimated by machine learning models, to *in vivo* data. Here, we refer to machine learning models as those performing multivariate calibration—a supervised regression model (e.g., principal components regression (PCR), partial least-squares regression (PLSR), elastic net, artificial neural network) is trained on voltammograms of known concentration to predict voltammograms of unknown concentration.^31^ The inability to deploy background-subtracted models trained *in vitro* (i.e., FSCV-PCR) to give consistent and reliable *in vivo* results has been demonstrated.^32−34^ This failure is, in part, thought to be due to the adsorption of interferents, especially metal cations and electro-inactive species such as proteins, which are rarely accounted for.^20^ No training paradigm can yet mimic the complex environment in the brain. However, even for a single analyte such as dopamine, a voltammetry technique paired with a suitable machine learning model that better bridges this *in vitro–in vivo* “generalization gap” would be extremely powerful; the state-of-the-art model in the field is moving toward this approach.^13,35−40^ Background-inclusive models appear to be a critical step in reducing the generalization gap due to the underutilized information content in background currents, as discussed by Movassaghi and co-workers.^11^

“As such, differences in the Helmholtz double layer, mass transport, analyte concentrations and adsorption, and other dynamic electrode surface properties occurring during an applied pulse are considered potential sources of analyte-specific information. This information is encoded in the transient responses of faradaic and non-faradaic currents. By including faradaic and non-faradaic current responses as input to the model (i.e., not background subtracting), the [model] selects aspects of the current response that covary with analyte identity and concentration. This is opposed to background-subtracted methods, where some information is discarded prior to model input to increase signal-to-noise. Potentially relevant information in the background is then lost.”—Movassaghi et al. 2021^11^

Statistical approaches to domain generalization, adaptation, and transfer learning offer promising improvements over classical chemometric validation techniques such as residual analysis.^31,37,38,41,42^ Nonetheless, some consider a barrier to the use of machine learning models in voltammetry the fact that the predictions can only be considered estimates until methods of ground-truth validation are possible. For neurochemical studies, *in vivo* experimental checks can inform predictive model selection and increase confidence and generalizability.^11^ These include confirming how analyte concentrations correlate with stereotaxic electrode positioning, stimulation frequency, pharmacology, behavior, and comparisons with other *in vivo* neurochemical methods, e.g., microdialysis.

### Dealing with Dynamics—Let the Machines Learn

Given the shortcomings of background subtraction described above, how should chemists and neuroscientists deal with background signal dynamics that impede generalization? A logical solution is background correction. However, background correction methods assume a temporally based parametric relationship within the signal that has the same issues of masking chemically interesting dynamics and can suffer similar pitfalls as background subtraction. A different approach to dealing with dynamic backgrounds is simply to train analysis models with the background current included (i.e., do not background subtract). Meunier et al. have shown several demonstrations.^15,24^

“The model, validated both in adrenal slice and live brain tissue, enables information encoded in the shape of the background voltammogram to determine electrochemical parameters that are critical for accurate quantification.”—Meunier et al. 2017^15^

**Figure 1:**
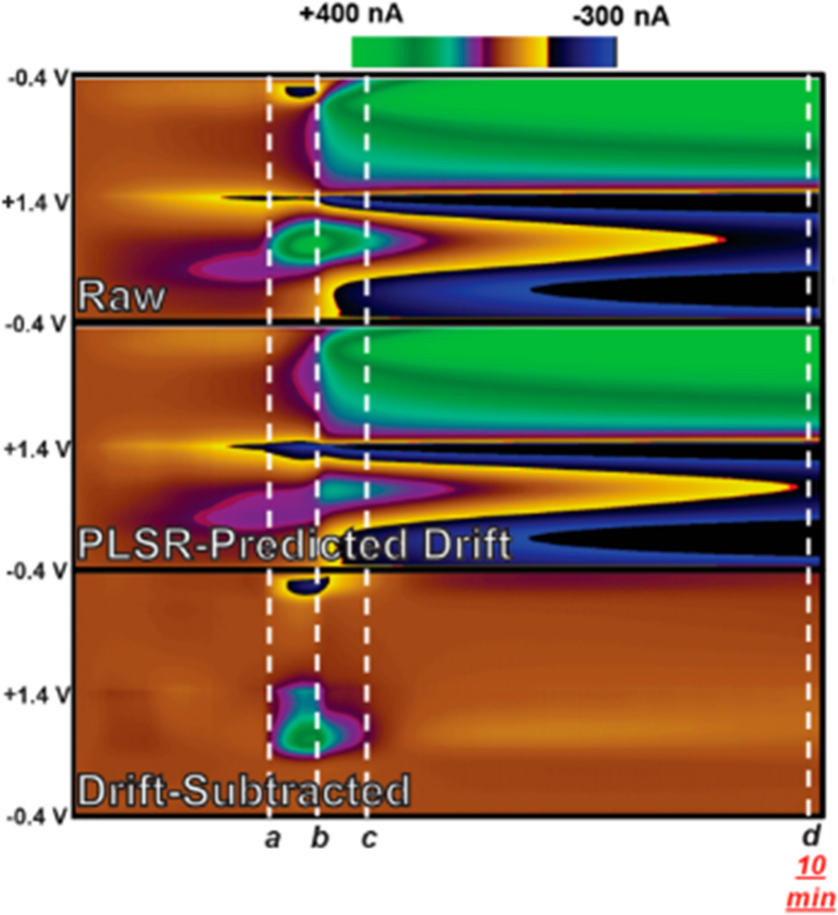
Predictive drift modeling generalizes *in vivo*. Reproduced from Meunier, C. J.; McCarty, G. S.; Sombers, L. A. *Anal. Chem*. **2019**, *91*, 7319–7327 (ref (24)). Copyright 2019, American Chemical Society.

Can machine learning models be effectively and accurately trained with dynamic backgrounds included? Or do dynamic backgrounds preclude the ability to obtain specific (i.e., trainable) electrochemical information? In machine learning terms, we aim to find a low-dimensional yet generalizable representation of the analytes, interferents, background current, irrelevant capacitive interference, etc. in the model. Sombers and co-workers have shown this is indeed possible, reporting a drift-prediction model that generalized across multiple electrodes (Figure 1).^24^

“Thus, it is clearly possible to develop effective models for subtraction of drift from fast voltammetric data that are not specific to any given electrode, to reveal both rapid and gradual changes in analyte concentration over time.”—Meunier et al. 2019^24^

Due to the prevalence of background subtraction for over three decades, suggesting its abstinence may seem controversial. Yet, in the past few years, avoiding background subtraction has been shown to be more reliable and robust for dopamine predictions than background-subtracted FSCV in the hands of experienced users.^11,30^ This is due to the application of modern machine learning methods that negate the need to use background subtraction to increase the signal-to-noise ratio. These pattern recognition algorithms are advanced enough to be trained on and to predict raw data extraordinarily accurately.

To lend additional credence to the idea of forgoing background subtraction, we point to studies in the mechanistic electrochemistry field. As opposed to using background-subtracted voltammograms to train machine learning models to predict analyte identity and concentration, fundamental electrochemistry studies use background-inclusive voltammograms to fit simulated and experimental data, including nonfaradaic current.^43−45^ These reports further demonstrate the utility of nonfaradaic information in models of electrochemical processes beyond concentration quantification. For example, areas of voltammograms not typically used in the manual assignment of electrochemical reaction mechanisms are now being used by deep learning classifiers for automated mechanistic assignment.^46^ Similar reports have emerged for fast voltammetry in terms of analyte quantification; *vide infra*.

**Figure 2:**
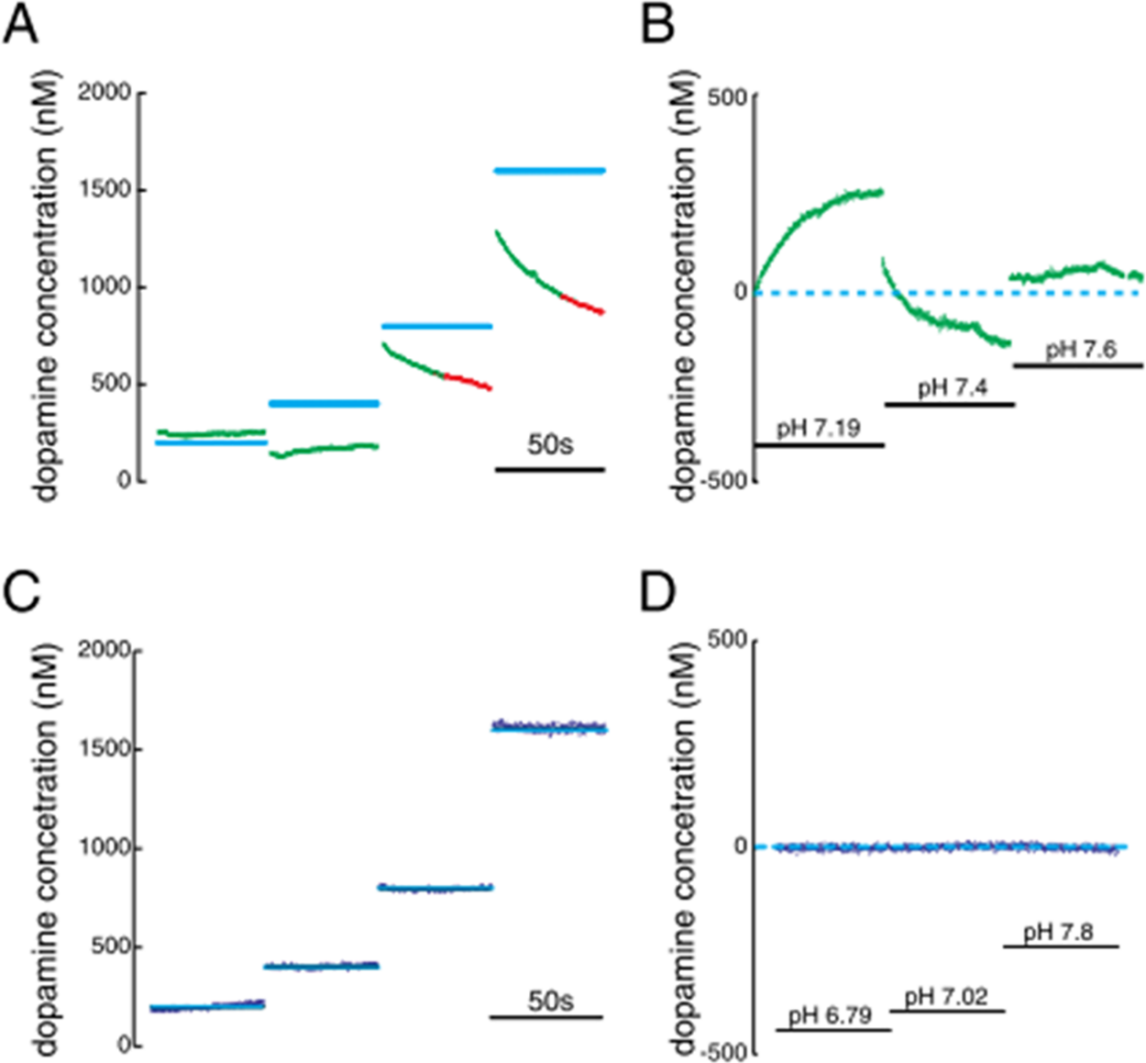
(A) Test set performance using an FSCV-PCR model trained on background-subtracted voltammograms for varying dopamine concentrations at pH 7.4 and (B) versus varying pH at constant dopamine (0 nM). (C,D) The same test set performance using an FSCV-elastic net model trained on nonbackground-subtracted data. Reproduced from Kishida, K. T.; Saez, I.; Lohrenz, T.; Witcher, M. R.; Laxton, A. W.; Tatter, S. B.; White, J. P.; Ellis, T. L.; Phillips, P. E. M.; Montague, P. R. *Proc. Natl. Acad. Sci. U.S.A.***2016**, *113*, 200–205 (ref (30)). https://creativecommons.org/licenses/by/4.0/.

The combined use of suitable supervised regression models and nonbackground-subtracted voltammograms as training data has been demonstrated repeatedly in recent literature to be more powerful than using background-subtracted data. For example, Kishida et al. showed that conventional background-subtracted FSCV-principal components regression (PCR) predictions were both unreliable for dopamine at low concentrations and confused changes in pH for dopamine, when compared to an elastic net model trained with the same nonbackground-subtracted data (Figure 2).^30^ Here, a pH change of 0.2 units resulted in a 250 nM dopamine prediction error (0 nM dopamine was present but 250 nM was predicted). Meanwhile, the nonbackground-subtracted data, when modeled, not only increased dopamine sensitivity (S/N ratio) but also did not confound pH for dopamine (roughly 0 nM dopamine was predicted for the same 0.2-unit pH change).

**Figure 3:**
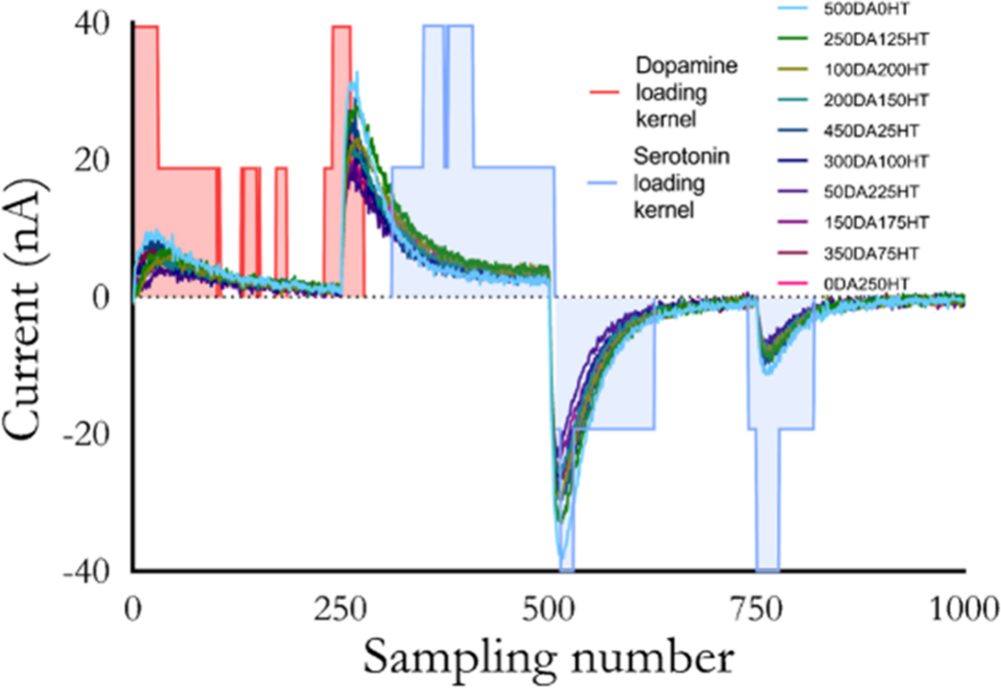
Model loadings analysis by analyte for rapid pulse voltammetry. Large loadings for dopamine and serotonin in the early portions of specific steps indicate the model is gaining analyte-specific information from portions of the current traces dominated by capacitive current. Reproduced from Movassaghi, C. S.; Perrotta, K. A.; Yang, H.; Iyer, R.; Cheng, X.; Dagher, M.; Fillol, M. A.; Andrews, A. M. *Anal. Bioanal. Chem*. **2021**, *413*, 6747–6767 (ref (11)). http://creativecommons.org/licenses/by/4.0/.

Importantly, a “good” signal-to-noise ratio as defined by the human eye, for example, following background subtraction, is not directly comparable to a “good” signal-to-noise ratio for a machine learning model where the signal-to-noise ratio is not based on the single-point, amplitude-based metric used for classical calibration curves. For machine learning models, entire voltammograms, each described by thousands of data points, are now being analyzed. The impact is demonstrated by nonbackground-subtracted data yielding higher sensitivity than background-subtracted data. Movassaghi et al. recently reported findings on the improved performance of background-inclusive models when compared directly to background-subtracted models.^11^ Further, Kishida et al. and Movassaghi et al. demonstrated that their models were using areas of the voltammograms normally discarded during background subtraction (i.e., nonfaradaic areas; Figure 3).^11,30^

Background subtraction can be thought of as a form of manual feature engineering useful for identifying oxidation and reduction peak currents for univariate linear regression, while multivariate models essentially perform automatic feature engineering. Thus, machine learning has overcome and surpassed the need for background subtraction as originally proposed. Rather than focusing on a small subset of the information in voltammograms, we can now utilize all voltammogram information.

One question is why nonbackground-subtracted techniques were not focused on sooner? For one, the resolution of previous generations of data acquisition cards was an impediment to approaches aimed at deconvoluting varying contributions of faradaic and nonfaradaic current.^2^ Data sampling speeds available today are an order of magnitude faster (i.e., <100 kHz vs >1 MHz). The increases in data density mean processes previously seen at 10-μs intervals are now captured at 1-μs intervals—the time scale of resolvable adsorptive/capacitive charging processes at carbon fiber microelectrodes (i.e., RC equivalent circuit-time constants of ∼4–40 μs have been demonstrated both empirically and theoretically).^21^

Moreover, large-scale and chemically diverse training sets were not and, generally, are still not utilized. Early reports of supervised regression models for background-subtracted fast voltammetry were trained solely on dopamine over a handful of concentrations and occasionally, a couple of metabolites at single concentrations, across dozens of voltammograms.^47^ The most advanced models today consist of far more robust experimental designs with training sets containing multiple concentrations of analytes, metabolites, H^+^ and other ions, multiple electrodes, and so on, across thousands of voltammograms.^38,48^ As state-of-the-art (i.e., deep learning) models are developed,^35,37,39,46^ electrochemists will also likely find greater success in maximizing the information content of data acquisition. Examples include the fusing of multiple data sources,^45^ the ability to perform inference on out-of-distribution data,^38^ and the use of physics-informed^43^ and probabilistic^49^ models. These areas are likely to yield complementary advances for machine learning and voltammetry that extend beyond neurochemical detection toward electroanalytical chemistry *writ large*.

While previous work has shown there is important information in the capacitive/nonfaradaic/background current, few methods have capitalized on background-inclusive models to improve analyte predictions. We surmise the future of fast voltammetry will rely increasingly on background-inclusive machine learning models because of the marked increases in performance associated with utilizing capacitive (nonfaradaic) current information. The latter is especially useful as an additional source of information for discriminating highly overlapping electrochemical signals, as shown for serotonin and dopamine (Figure 3).^11,48^ Adsorption, interfacial surface chemistry, drift, and other contributions all affect capacitive, in addition to faradaic currents. Subtracting the background removes relevant information that mathematical algorithms can use for more robust training and thus more accurate predictions. In addition to improvements in sampling, better digital electronics and data acquisition cards can now be used to drive more rapid potential changes with high slew rates.

### Waveform Woes: Powerful Pulses or Skillful Sweeps?

The pulse versus sweep waveform debate has permeated the history of voltammetry (much like an earlier debate between the “sparks” and the “soups” regarding the nature of communication at synapses^50^). Osteryoung advocated as early as 1993 for a “pulse revolution”, suggesting that progress in electronics and computing would advance pulse voltammetry in a postmodernist era.^51^ Ironically, prior to FSCV adoption, electroanalytical chemists avoided fast cyclic voltammetry because of the large background currents generated by fast sweeps. Once background subtraction appeared to alleviate issues associated with large and temporally evolving background currents in FSCV, the use of pulse techniques fell by the wayside because of their slow temporal resolution (associated with differential sampling between nonfaradaic and faradaic currents and slow electronics).^2^ However, if the background current is indeed no longer an issue and is a rich source of information, then electroanalytical chemists are free to explore the use of pulse waveforms 30 years after Osteryoung’s prediction.

“Although the principles of capacitive and faradic current had already been widely known, the straight nature of [pulse voltammetry], where it is easy to separate capacitive and faradic current, has been overlooked, and not utilized for voltammetric recordings in the brain.”—Yoshimi et al. 2014^59^

Both sweep and pulse waveforms enable users to customize start and stop potentials for different waveform segments. Sweep techniques offer customizable scan rates, whereas pulse techniques allow customizable step potentials and hold times. In fact, a digitally generated sweep signal is a series of small pulses. One argument against sweep voltammetry is that variable scan rates do not provide a different type of fundamental chemical information. That is linear scans (sweeps) inextricably link time with potential and faradaic with capacitive current. In any case, variable scan rates,^52^ multiple scan rates,^53^ and multisweep voltammetry methods^54−57^ have been developed.

In theory, pulsed voltammetry provides fundamentally distinguishable faradaic and nonfaradaic information, whereas fast-sweep voltammetry does not. In the latter, the capacitive current is rapidly evolving throughout the waveform, making it difficult to separate faradaic from nonfaradaic current contributions. Nonetheless, these different sources of current need not be separated to be practically accurate or useful for quantifying an analyte (although, formally modeling these separate contributions can be useful for other tasks, such as equivalent circuit models^58^). In step potentials, even for fast steps, the full capacitive decay profiles (change in current over the step time) provide information to parse capacitive and faradaic current contributions. Yoshimi et al. were one of the first to demonstrate that a single rectangular pulse could differentiate dopamine and pH, even in the presence of serotonin and ascorbate, solely by changes in the capacitive current response, without explicit training sets.^59^ Meanwhile, dopamine and pH predictions were confounded in FSCV.

**Figure 4:**
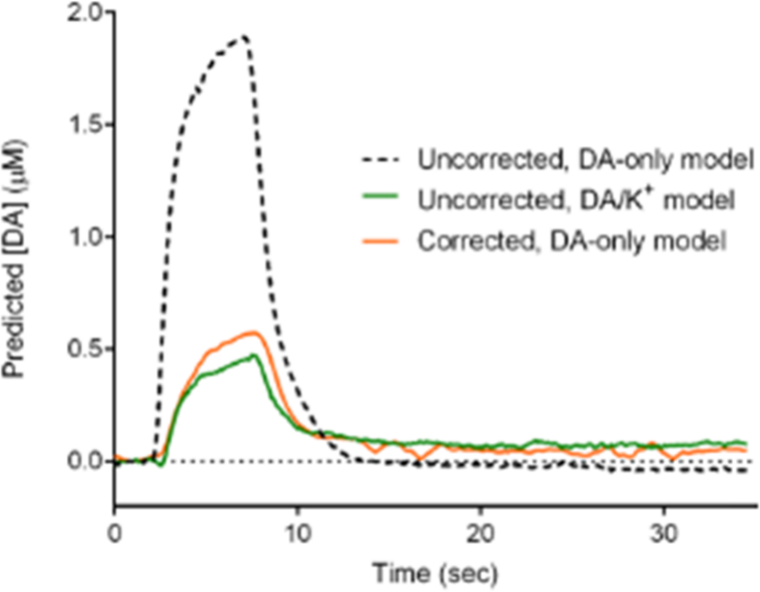
Dopamine (DA) predictions from FSCV data containing 120 mM K^+^ for an actual value of 500 nM dopamine. Reproduced from Johnson, J. A.; Hobbs, C. N.; Wightman, R. M. *Anal. Chem*. **2017**, *89*, 6166–6174 (ref (21)). Copyright 2017, American Chemical Society.

Following this work, Wightman and co-workers, who originally promulgated background subtraction in FSCV, reported a convolution-removal technique for the oft-ignored contributions from monovalent ions (K^+^, Na^+^),^21^ and extended this thinking to divalent cations (Mg^2+^, Ca^2+^).^60^ For example, background-subtracted FSCV-PCR confused a 120 mM change in [K^+^] as a 1.5 μM change in dopamine, when no actual change in dopamine occurred.^21^ Only when the PCR model was trained across [K^+^] or when the deconvolution technique was used did the model not confuse K^+^ for dopamine (Figure 4). However, training a model across [K^+^] requires repeating the original training set while varying [K^+^], increasing in training times and samples. The deconvolution technique required another computation step and augmentation of the waveform and has only been tested for the case of a single analyte. Interestingly, this deconvolution method relies on a small amplitude *pulse* integrated with the FSCV *sweep* to separate the expected capacitive ionic current.

**Figure 5:**
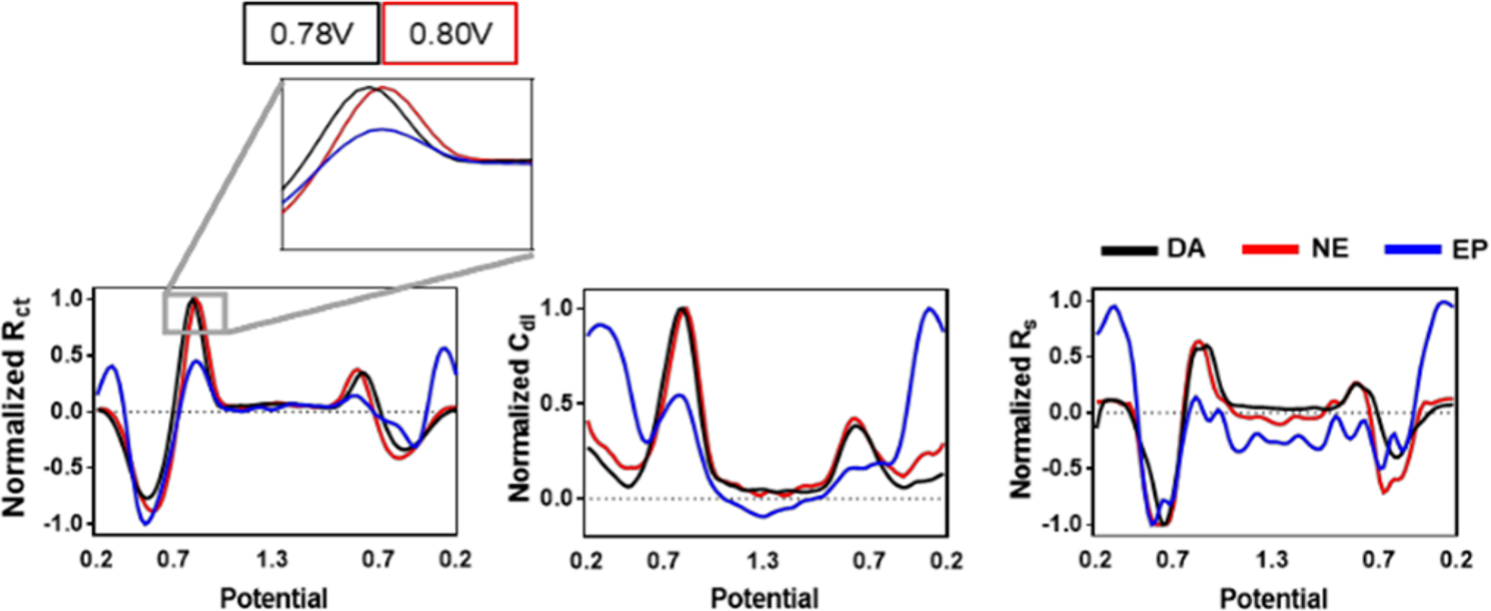
Analyte-specific equivalent circuit voltammograms for dopamine (DA), norepinephrine (NE), and epinephrine (EP). Reproduced from Park, C.; Hwang, S.; Kang, Y.; Sim, J.; Cho, H. U.; Oh, Y.; Shin, H.; Kim, D. H.; Blaha, C. D.; Bennet, K. E. *Anal. Chem*. **2021**, *93*, 15861–15869 (ref (14)). Copyright 2021, American Chemical Society.

Sweep waveforms remain widely used. Sweeps contain important information in their backgrounds, as shown for nonbackground-subtracted elastic net FSCV.^13^ Nonbackground-subtracted FSCV paired with elastic net analysis has been used to decode dopamine and serotonin signaling in human striatum involved in decision making.^9,30,48^ Moreover, deep learning algorithms have been used with nonbackground-subtracted FSCV to determine subsecond norepinephrine signals in human amygdala associated with the emotional regulation of attention.^40^ Sombers and co-workers have not only reported on the ability of FSCV with machine learning to predict voltammetric drift^24^ and the usefulness of background voltammograms as accurate experimental parameter predictors,^61^ they explored the impedance (i.e., capacitive) characteristics of electrodes and analyte-containing solutions through electrochemical impedance spectroscopy (EIS).^62,63^ Similarly, later work by the Jang group advocated for modeling analyte-specific equivalent circuit parameters (Figure 5) and utilizing double-layer capacitance as a feature to improve biofouling robustness.^14^ This work used a novel pulse voltammetry technique.

Using only square wave voltammetry (SWV), Cobb and Macpherson showed that circuit parameters can be extracted directly from the nonfaradaic regions in SWV, circumventing the need for EIS.^64^ Circuit parameters can then be used to differentiate responses unique to electrolyte vs. analyte concentration dynamics or serotonin biofouling. *In vivo* voltammetry experiments are plagued by the confounding factors of unknown electrolyte composition dynamics and surface biofouling. The nonfaradaic information contained within pulses has direct utility in addressing this aspect of the generalization gap (*vide supra*).

“The SWV capacitance data can be used to provide real time monitoring on whether a changing faradaic signal is due to concentration changes of the electrochemically active analyte or fouling of the electrode.”—Cobb and Macpherson 2019^64^

**Figure 6:**
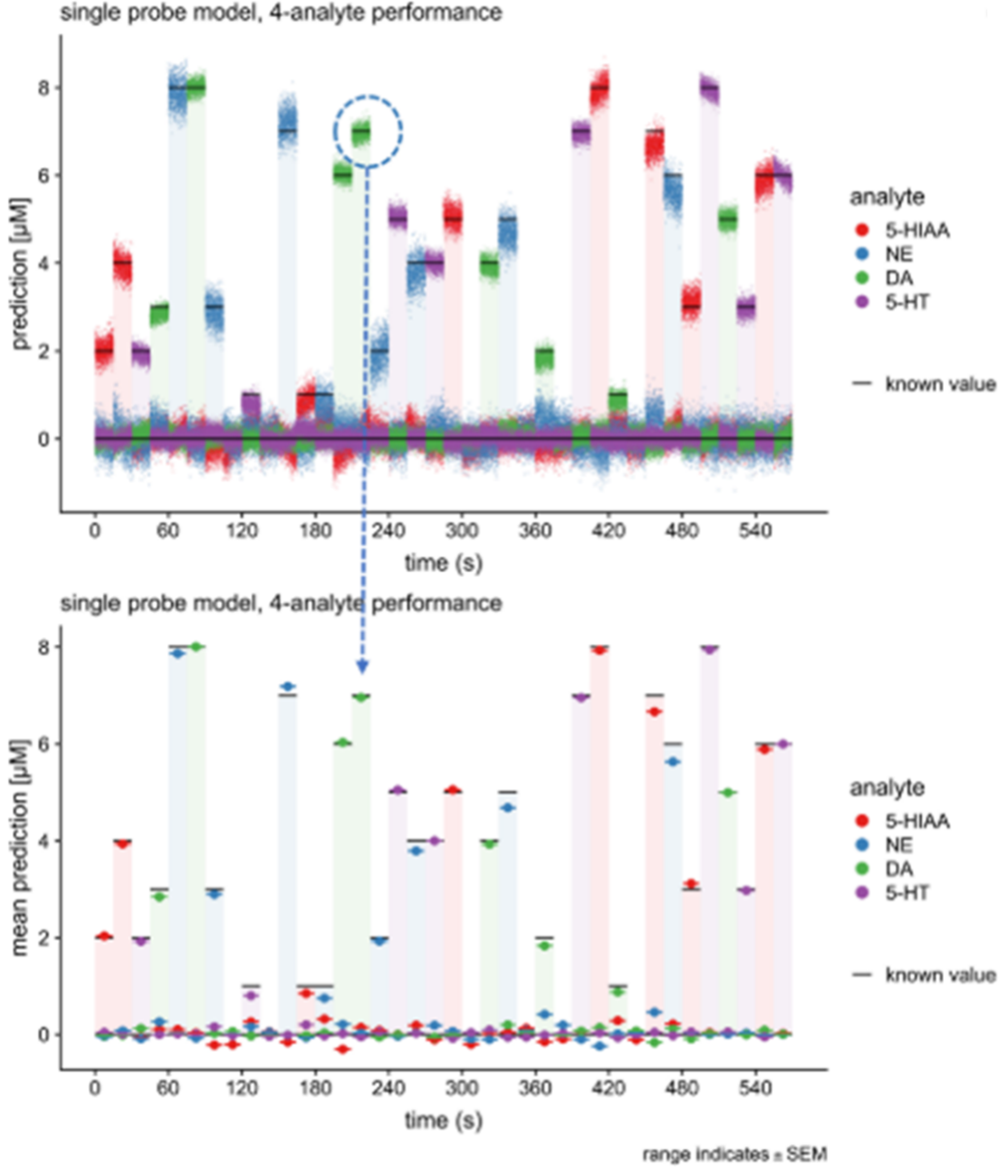
Analyte (dopamine (DA), norepinephrine (NE), serotonin (5-HT), and 5-hydroxyindoleacetic acid (5-HIAA)) predictions from randomized pulse voltammetry. Reproduced with permission from Montague, P. R.; Lohrenz, T.; White, J.; Moran, R. J.; Kishida, K. T. *bioRxiv Preprint*, 2019 (ref (65)). Copyright 2019, The Authors.

The studies discussed above advocate for the utility of pulse voltammetry, beyond its being complementary to FSCV. Moreover, two methods to date on background-inclusive, customized, rapid or “burst” pulses have both achieved detection of notoriously difficult analyte mixtures, i.e., codetection of dopamine and serotonin,^11^ and dopamine and norepinephrine (Figure 6).^65,66^ Rapid pulse voltammetry was also used to demonstrate the first evidence of combined measurements of basal neurotransmitter levels and stimulated release via a single technique.^11^ Outside of bioanalytical voltammetry, the usefulness of pulse-induced capacitive current has been demonstrated repeatedly and is becoming more commonplace as advanced data acquisition and analysis speeds enable its exploration. The voltammetric electronic tongue community has recognized the importance of modeling information contained in nonfaradaic current, in addition to faradaic current, for decades, especially in complex environments.^67−70^ The high information content of pulses and their accessible capacitive currents is also gaining attention for electrochemical measurements in other fields.^71,72^

For neurochemical analyses, the debate on pulse versus sweep waveforms is expected to continue. While neurochemical fast voltammetry has been tailored toward sweeps, pulses offer relatively unexplored information and use cases. Some have advocated for the complementary use of separate pulse and sweep waveforms (i.e., data fusion),^59^ while the Heien, Jang, and Lee groups have pioneered techniques that combine sweeps and pulses into single waveforms.^6,73−75^ Others have concatenated pulse and sweep waveforms for a variety of electrochemical detection purposes.^76,77^ Approaches outside of the DC-realm (i.e., AC-voltammetry)^78^ are also garnering a resurgence of interest when combined with machine learning.^44,79^ Regardless of waveform type, we propose that nonbackground-subtracted approaches are well suited to facilitate the union of voltammetry and machine learning due to the importance of including the capacitive current in the training sets.

To extract maximal neurochemical information from the brain, we recommend that voltammetry practitioners extract maximal information from their data to provide information on absolute vs relative changes in stimulated neurotransmitter levels, basal neurotransmitter levels, and simultaneous analyte monitoring. Based on the publications reviewed here on the importance of nonfaradaic information and the versatility of waveforms (sweeps and pulses) in voltammetry, the next major advances for *in vivo* voltammetry appear likely to come from background-inclusive approaches paired with machine learning. There are many recent examples of movement in this direction inside and outside of the chemical neuroscience community.^11,14,60,21,44−46,65,66,72,73,75,80^

If there is a solution to the pervasive problems that have plagued voltammetry for decades preventing the full electrochemical exploration of the chemical communication systems of the brain and beyond, recent evidence points to a need to reconsider the use of background subtraction. Broadly speaking, all practitioners of voltammetry should consider maximizing the information inherent in their experimental data and complementing domain knowledge with their analysis toolkit of choice.

“There is scientific value to capturing more current data generated during square wave voltammetry...it contains valuable information about the double layer charging and interfacial processes occurring at short time scales. More specifically...analyzing the current-time data from the non-Faradaic region of the potential pulse can provide crucial information.”—Abeykoon et al. 2023^72^
